# Anterior Interosseous Nerve to Ulnar Motor Nerve Transfer for High Ulnar Nerve Injuries: A Prospective Pilot Case Series from a Resource-Limited Setting

**DOI:** 10.1055/a-2977-6887

**Published:** 2026-11-13

**Authors:** Talha Humayun, Ceemal F. Khan, Touqeer Hussain, Safdar A. Shaikh, Sobia Yasmeen, Mohammad F. Rahman

**Affiliations:** 1 Department of SurgeryAga Khan UniversityKarachiSindhPakistan

**Keywords:** ulnar nerve injury, anterior interosseous nerve, nerve transfer, intrinsic hand muscles, motor recovery, resource-limited setting

## Abstract

**Background:**

High ulnar nerve injuries result in profound intrinsic hand dysfunction, and conventional reconstructive options often yield suboptimal outcomes. Transfer of the anterior interosseous nerve (AIN) to the deep branch of the ulnar motor nerve (DBUMN) has emerged as a promising alternative. However, evidence from low- and middle-income countries remains limited.

**Methods:**

A prospective case series was conducted at a tertiary care center between January 2021 and May 2023. Six patients with high ulnar nerve injuries underwent end-to-end AIN to DBUMN nerve transfer. Outcomes were assessed using the British Medical Research Council (BMRC) grading system, grip strength, and functional status using a structured questionnaire at 1 year postoperatively. Correlation between time to surgery and motor recovery was analyzed using Spearman's rank correlation.

**Results:**

The mean age was 26.2 ± 15.7 years, and the mean time from injury to surgery was 5.2 months. At 1 year, intrinsic muscle strength improved from BMRC grade 0 preoperatively to a mean of 4.1. Mean grip strength reached 51.3% of the contralateral side. Earlier surgical intervention was associated with better motor recovery (Spearman correlation, exploratory analysis, *p* = 0.016). Most patients reported functional independence with mild to moderate difficulty in fine motor tasks.

**Conclusion:**

AIN-to-DBUMN transfer demonstrates promising early functional recovery in high ulnar nerve injuries. Outcomes appear to be influenced by timing of intervention. These findings support the feasibility of this technique in resource-limited settings, although larger studies are required to validate these results.

**Level of Evidence:**

Level IV.

## Introduction

High ulnar nerve injuries are associated with significant functional morbidity due to loss of intrinsic hand muscle function, resulting in impaired dexterity, reduced grip strength, and characteristic clawing deformity of the ulnar digits. 1 2 Conventional reconstructive strategies, including nerve repair, grafting, and tendon transfers, often yield suboptimal and non-physiological outcomes, particularly in proximal injuries. 3

Nerve transfer techniques have gained increasing attention as a means of restoring motor function by reinnervating distal targets using expendable donor nerves. The anterior interosseous nerve (AIN), a motor branch of the median nerve, is a suitable donor due to its redundancy in forearm function. 4 Transfer of the AIN to the deep branch of the ulnar motor nerve (DBUMN) allows targeted reinnervation of intrinsic hand muscles, which are essential for fine motor control.

Several studies have demonstrated favorable outcomes following AIN to DBUMN transfer, including improvements in grip strength and motor recovery. 5 6 7 However, most available evidence originates from high-income settings, where access to advanced rehabilitation services may influence outcomes. Data from low- and middle-income countries (LMIC) settings remain limited, where delayed presentation, high-energy trauma, and restricted rehabilitation resources may impact recovery.

This study presents a prospective pilot case series evaluating functional outcomes following AIN to DBUMN transfer in a resource-limited setting, with particular emphasis on motor recovery and timing of intervention.

## Materials and Methods

### Study Design and Setting

This prospective case series was conducted at a tertiary care hospital in Karachi between January 2021 and May 2023. Ethical approval was obtained (IRB: 2024-10398-30153), and informed consent was obtained from all participants.

### Patient Selection

Patients were eligible for inclusion if they met the following criteria:

**Table:** 

Traumatic high ulnar nerve injury	Complete intrinsic motor deficit (BMRC grade 0)	Diagnosis confirmed using electrodiagnostic studies

Patients were excluded if they had

**Table:** 

Prior reconstructive procedures for ulnar nerve injury	Associated injuries affecting hand function	Incomplete follow-up data

Preoperative assessment in all six patients confirmed normal median nerve and AIN motor function (intact pronator quadratus and flexor pollicis longus/flexor digitorum profundus to the index finger on clinical examination, corroborated by electrodiagnostic studies), confirming donor nerve suitability prior to transfer. Patients with associated injuries affecting hand function were excluded a priori to reduce confounding of the motor and functional outcomes reported here.

### Outcome Measures

Patients were followed at 3-month intervals for 1 year.

Primary outcome: Motor recovery using the British Medical Research Council (BMRC) grading.
Secondary outcomes: Grip strength via percentage of contralateral hand and functional status were assessed using a structured activities-of-daily-living questionnaire.

The activities-of-daily-living questionnaire was a non-validated instrument developed by the study team for the purpose of this pilot series, comprising seven task-specific items rated on a 5-point difficulty scale (1 = no difficulty, 5 = unable to perform). It was administered by the treating surgical team as a structured interview at the 1-year follow-up visit. As it has not undergone formal validation or psychometric testing, findings based on this instrument should be regarded as descriptive rather than definitive.

Motor recovery of the ulnar intrinsic musculature was graded using the BMRC scale, applied specifically to the first dorsal interosseous, abductor digiti minimi, and adductor pollicis (the latter assessed clinically via Froment sign). Intrinsic function was tested in isolation from the long flexors and extensors, which were innervated proximal to the level of injury and were confirmed to be intact preoperatively in all patients.

### Statistical Analysis

Descriptive statistics were reported as mean ± standard deviation. Spearman's rank correlation was used to assess the relationship between time to surgery and motor recovery. A *p* -value <0.05 was considered statistically significant.

Given the small sample size ( *n* = 6), correlation analyses were considered exploratory and hypothesis-generating rather than confirmatory, and results should be interpreted with caution.

### Surgical Technique

All procedures were performed under general anesthesia without muscle relaxation. A distal forearm incision was made to expose Guyon's canal and the ulnar nerve. The deep motor branch of the ulnar nerve was identified through intraneural dissection and traced proximally. In the proximal forearm, the AIN was identified at the level of the pronator quadratus and confirmed using intraoperative stimulation. The AIN was divided distally and transferred to the motor component of the ulnar nerve. Tension-free end-to-end coaptation was performed using 8–0 polypropylene sutures, supplemented with fibrin glue. The wound was closed in layers, and the wrist was immobilized in a neutral position for 3 weeks, followed by structured physiotherapy.

The rehabilitation protocol consisted of 3 weeks of wrist immobilization in a neutral splint, followed by supervised physiotherapy incorporating passive and active range-of-motion exercises, intrinsic muscle re-education, and sensory re-education. Therapy was delivered twice weekly for the first 3 months and then tapered according to individual progress, supplemented by a structured home exercise program. Motor re-education emphasized isolated activation of the ulnar intrinsic muscles to promote cortical adaptation following the nerve transfer.

## Results

A total of six male patients with traumatic high ulnar nerve injuries underwent end-to-end AIN to DBUMN transfers between January 2021 and May 2023. The mean age at surgery was 26.17 ± 15.70 years (range: 12–53 years). The mechanisms of injury included gunshot wounds ( *n* = 2), bomb blast injuries ( *n* = 2), and glass lacerations ( *n* = 2). All patients presented with ulnar claw hand and were right-hand dominant, with five of the six injuries involving the right hand ( Table 1 ).

**Table 1:** Patients characteristics

Patient	Age (years)	Sex	Mechanism	Side	Dominant hand	Time to surgery (months)	Follow-up (months)
1	33	Male	Gunshot	Left	Right	4	16
2	17	Male	Bomb blast	Right	Right	4	18
3	53	Male	Bomb blast	Right	Right	5	14
4	13	Male	Glass laceration	Right	Right	6	15
5	29	Male	Glass laceration	Right	Right	8	14
6	12	Male	Gunshot	Right	Right	4	19

Note: The mean interval from injury to surgical intervention was 5.17 ± 1.60 months (range: 4–8 months). The mean postoperative follow-up duration was 16.0 ± 2.09 months.

Preoperatively, all patients had a BMRC motor grade of 0 in the intrinsic muscles of the affected hand. At 1-year follow-up, the mean BMRC grade improved to 4.1, indicating substantial motor recovery across the cohort ( Fig. 1 ). Patients who underwent surgery earlier (≤4 months) tended to demonstrate better functional outcomes with earlier and stronger intrinsic muscle reinnervation. Spearman correlation suggested an inverse relationship between delay to surgery and final motor outcome (exploratory analysis, *p* = 0.016). Grip strength, measured using a sphygmomanometer at 1 year postoperatively, revealed that the operated hand achieved an average of 51.33% of the contralateral (unaffected) hand's strength ( Fig. 2 ). The highest grip strength recovery was observed in patients who had surgery within 4 months of the injury.

**Fig. 1:**
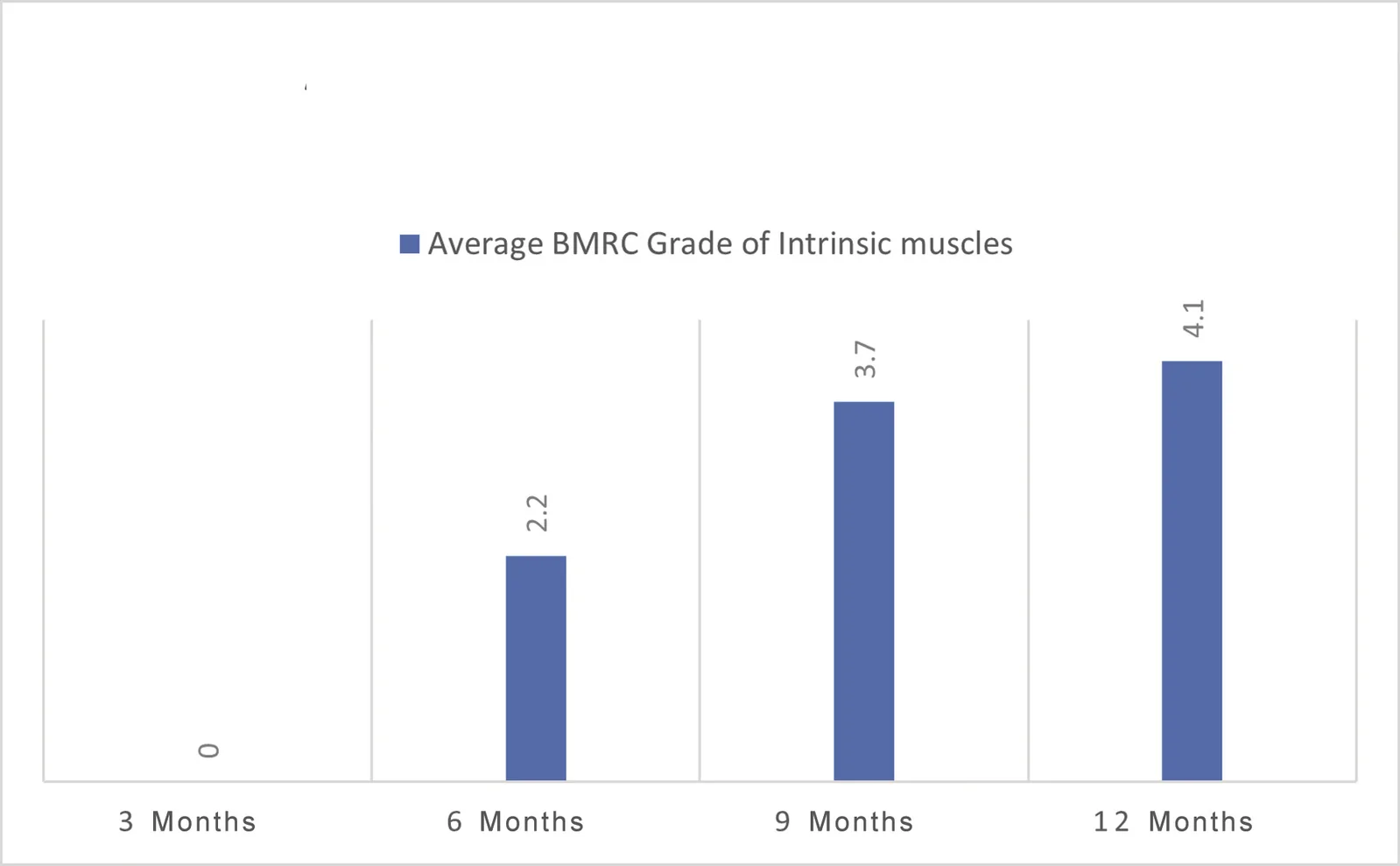
Mean British Medical Research Council (BMRC) grade of the ulnar intrinsic muscles of the affected hand at each postoperative follow-up interval (preoperative, 3, 6, 9, and 12 months), illustrating the trajectory of motor recovery following AIN-to-DBUMN transfer. Error bars represent standard deviation.

**Fig. 2:**
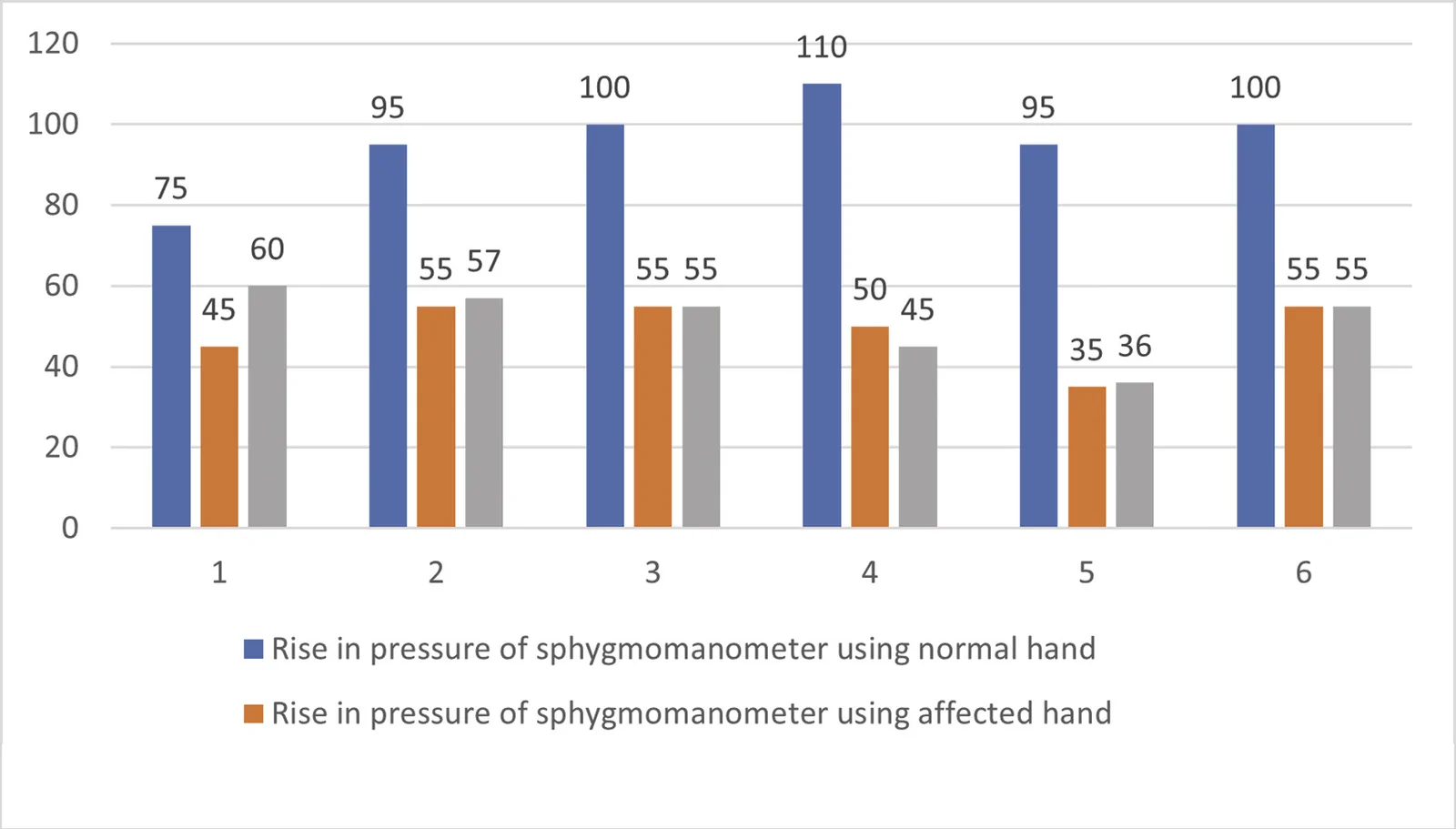
Grip strength of the operated hand at 1-year follow-up, expressed as a percentage of the contralateral (unaffected) hand for each of the six patients, measured using a sphygmomanometer.

At final follow-up, residual clawing, Froment sign, and Wartenberg sign were reassessed in all six patients as markers of intrinsic muscle balance. Residual clawing persisted in five out of six patients, Froment sign was positive in six out of six patients, and Wartenberg sign was present in five out of six patients.

To assess the impact of motor recovery on functional autonomy, patients completed a structured questionnaire rating difficulty in seven daily activities using the affected hand. Tasks included opening jars, turning doorknobs, holding objects, buttoning shirts, writing, cutting food, and unlocking doors. The majority of patients reported mild to moderate difficulty in performing these tasks, with mean difficulty scores ranging from 1.8 to 3.2 across activities ( Table 2 ). Despite persistent challenges in tasks requiring fine motor precision, most patients achieved functional independence and expressed satisfaction with the surgical outcomes.

**Table 2:** Ability to perform the following activities of daily living as rated by patients using the affected hand (using scale: 1 = no difficulty, 5 = unable to perform)

S. No.	Opening a tight jar	Turning a doorknob	Holding a glass/cup without spilling	Buttoning a shirt	Writing with a pen	Cutting food with a knife	Unlocking a door lock using a key
1	1	2	3	2	4	1	3
2	2	2	3	2	2	3	2
3	3	2	3	2	3	3	2
4	3	3	2	2	2	2	2
5	2	2	4	3	5	3	5
6	1	2	1	3	2	2	3

Note: Overall, five out of six patients reported being satisfied with the results of the procedure and would recommend it to others. The procedure yielded consistent motor recovery and functional improvements across the cohort, reinforcing its feasibility and effectiveness even in a resource-limited setting.

## Discussion

This prospective pilot study suggests that AIN-to-DBUMN transfer may achieve meaningful motor recovery in patients with high ulnar nerve injuries, even within a resource-limited health care setting. Improvements in intrinsic muscle strength and functional independence were observed, although limitations in fine motor precision persisted.

The degree of recovery observed in this study is consistent with previously reported outcomes. Sallam et al reported M3–M4 recovery in the majority of patients undergoing nerve transfer procedures, 8 while Chen et al demonstrated significant improvements in motor function and grip strength following AIN to DBUMN transfer. 9 Similarly, Gross et al reported improved muscle function, although complete restoration of normal hand function was not achieved. 10 Residual deficits in fine motor tasks may be explained by axonal mismatch between the donor and recipient nerves. Anatomical studies have demonstrated a lower axon count in the AIN relative to the DBUMN, which may limit full reinnervation. 11 Furthermore, incomplete motor unit reinnervation below a critical threshold may result in suboptimal force generation. 12

An important observation in this study was the association between earlier surgical intervention and improved motor recovery. This aligns with existing evidence highlighting the importance of timely nerve transfer in optimizing outcomes. The end-to-end AIN-to-DBUMN transfer used in this series differs from the supercharged end-to-side (SETS) variant described by Chen et al, 9 in which the AIN is coapted to the DBUMN without dividing the native ulnar motor fascicles, preserving the potential for spontaneous ulnar reinnervation alongside the transferred axons. The SETS technique is generally favored when some residual or recovering ulnar motor function is anticipated, or when the interval to surgery is short enough that native axonal regeneration remains plausible, as it does not sacrifice this possibility. Conversely, an end-to-end transfer, as performed in the present series, may be preferable in patients with complete (BMRC grade 0) and long-standing intrinsic paralysis, where reliance on native ulnar regeneration is unlikely and maximizing axonal input to the DBUMN through a full end-to-end coaptation may be advantageous. Reported outcomes between the two techniques are broadly comparable, though direct comparative data remain limited, and the choice between end-to-end and SETS transfer in practice is often guided by time from injury, findings on intraoperative stimulation of the native ulnar motor fascicles, and surgeon preference rather than by high-level comparative evidence.

## Strengths

This study highlights the feasibility of performing advanced nerve transfer procedures in LMIC settings. Patients in this cohort frequently presented with high-energy trauma and had limited access to specialized rehabilitation services. Despite these constraints, functional outcomes were encouraging, supporting the applicability of this technique beyond high-resource environments.

## Limitations

This study has several limitations. The small sample size and single-center design limit generalizability. The absence of a control group and reliance on contralateral comparison may introduce bias. Functional outcomes were partly subjective. These findings should therefore be interpreted as preliminary.

Postoperative electrophysiological studies to objectively document reinnervation were not performed in this series; outcome assessment relied on clinical motor grading, which may be subject to observer variability. Postoperative electrodiagnostic studies were not performed in this series. The functional questionnaire used to assess activities of daily living was developed in-house and has not been validated, and no validated patient-reported outcome measure was used; this, combined with the small sample size, limits the statistical power of this study and the generalizability of its exploratory associations, including the observed relationship between timing of surgery and motor recovery.

## Conclusion

AIN-to-DBUMN nerve transfer shows promising early functional outcomes in patients with high ulnar nerve injuries. Earlier intervention may be associated with improved recovery. These findings support the feasibility of this technique in resource-limited settings; however, larger controlled studies are required to confirm these results.
